# Ten things to get right for marine conservation planning in the Coral Triangle

**DOI:** 10.12688/f1000research.3886.3

**Published:** 2015-12-21

**Authors:** Rebecca Weeks, Robert L. Pressey, Joanne R. Wilson, Maurice Knight, Vera Horigue, Rene A. Abesamis, Renerio Acosta, Jamaluddin Jompa

**Affiliations:** 1 Australian Research Council Centre of Excellence for Coral Reef Studies, James Cook University, Townsville, Australia; 2 Sea Solutions, Pottsville, Australia; 3 USAID Coral Triangle Support Partnership, Jakarta, Indonesia; 4 Silliman University Angelo King Center for Research and Environmental Management, Dumaguete, Philippines; 5 USAID Regional Development Mission for Asia, Bangkok, Thailand; 6 Department of Marine Science, Hasanuddin University, Makassar, Indonesia

**Keywords:** conservation, coral, reef, Coral Triangle

## Abstract

Systematic conservation planning increasingly underpins the conservation and management of marine and coastal ecosystems worldwide. Amongst other benefits, conservation planning provides transparency in decision-making, efficiency in the use of limited resources, the ability to minimise conflict between diverse objectives, and to guide strategic expansion of local actions to maximise their cumulative impact. The Coral Triangle has long been recognised as a global marine conservation priority, and has been the subject of huge investment in conservation during the last five years through the Coral Triangle Initiative on Coral Reefs, Fisheries and Food Security. Yet conservation planning has had relatively little influence in this region. To explore why this is the case, we identify and discuss 10 challenges that must be resolved if conservation planning is to effectively inform management actions in the Coral Triangle. These are: making conservation planning accessible; integrating with other planning processes; building local capacity for conservation planning; institutionalising conservation planning within governments; integrating plans across governance levels; planning across governance boundaries; planning for multiple tools and objectives; understanding limitations of data; developing better measures of progress and effectiveness; and making a long term commitment. Most important is a conceptual shift from conservation planning undertaken as a project, to planning undertaken as a process, with dedicated financial and human resources committed to long-term engagement.

## Introduction

The Coral Triangle, which encompasses the marine waters of Indonesia, Malaysia, Papua New Guinea, Philippines, Solomon Islands, and Timor-Leste, is the epicentre of marine biodiversity and widely recognised as a global conservation priority ^1^. In addition to their conservation value, the Coral Triangle’s marine resources are a cornerstone of the region’s economies and societies, with millions of people dependent upon them as a daily source of food and income ^2^. The health of these ecosystems is at severe risk due to destructive and over-fishing, coastal development, poor water quality, and climate change ^3^.

In 2009, the six Coral Triangle countries, supported by USAID and other external funders, embarked upon the Coral Triangle Initiative on Coral Reefs, Fisheries and Food Security (CTI-CFF), an unprecedented multilateral partnership to address threats to the region’s marine and coastal resources through accelerated and collaborative action. The CTI-CFF goals include the designation of priority seascapes, establishment of a Coral Triangle marine protected area (MPA) system, the protection of threatened species, coordinated action on climate adaptation, and implementation of an ecosystem approach to fisheries management ^4^. As the initial five-year phase of the CTI-CFF came to a close in 2013, much has been accomplished, but much work remains if these goals are to be achieved ^5^.

Marine conservation planning is a systematic approach to developing spatial plans, primarily focused on conservation of biodiversity, habitats and ecological processes, while facilitating multiple uses of the marine environment and promoting, where possible, goals related to climate change, fisheries, and livelihoods. In the last three decades, conservation planning has evolved from an academic discipline to have considerable influence on conservation action around the world ^6^. Yet, systematic approaches have had relatively little influence on conservation in the Coral Triangle – one region where they are needed most.

Conservation planning faces particular challenges in the Coral Triangle. The marine, coastal, and small-island ecosystems that are the focus of the CTI-CFF comprise large-scale common pool resources, which are notoriously difficult to govern ^59^. The Coral Triangle is characterised by extremely diverse political, economic, and cultural contexts, and conservation planning must therefore contend with a huge diversity of institutional settings, objectives and social and political actors operating at multiple levels and scales ^59^. Nevertheless, whilst applications might be less straightforward than in simpler institutional settings, the potential benefits of conservation planning are substantial.

Conservation planning provides benefits at both regional and local scales. Regional-scale planning is critical for achieving objectives that require broad perspectives and emergent properties ^7^ that mean the whole (e.g. a system of MPAs) is greater than the sum of the parts (individual MPAs). Emergent properties are achieved through, for example, complementarity of ecosystems and species and connectivity between individual MPAs ^8, 9^. Systematic planning provides a transparent framework to ensure efficient use of limited resources, and offers a proactive alternative to reactive actions in the face of increasing threats to natural resources ^10, 11^. Importantly, systematic planning can be used to minimise conflict between conservation goals and the diverse aspirations of users of the marine environment ^12, 13^.

The Coral Triangle has experienced rapid growth in the number of individual MPAs designated or initiated primarily by communities and local governments. Yet, despite the many benefits of local and community-led actions, few MPAs are effectively managed ^14^ and, without coordination, they often fail to form functional conservation networks that achieve regional-scale objectives ^15– 17^. Systematic planning can inform strategic expansion of local actions to maximise their cumulative contribution towards regional-scale goals ^18, 19^, including the broad goals of the CTI-CFF.

With the first phase of the CTI-CFF now complete, it is timely to explore challenges related to the effective implementation of conservation planning in the region, and how they might be overcome. To do so, we convened a focus group of conservation biologists, practitioners, policy makers, and donors working in the region (the authors). We first outlined a vision: of conservation planning applied throughout the Coral Triangle, at spatial scales ranging from local to region-wide, to effectively inform management actions implemented to achieve objectives for biodiversity, fisheries, and food security. We then considered constraints on this vision being realised, and sought to identify strategies to overcome the constraints.

From an initial list of “things to get right”, we consolidated related topics, and excluded those that we considered either trivial or overly specific, to arrive at the final 10 ( Table 1). We focussed specifically on challenges related to the uptake and application of conservation planning, as opposed to those facing conservation initiatives more generally. The topics that we discuss here are deliberately ambitious, and we do not claim either a complete analysis of these challenges or to provide solutions. Our aim is to highlight issues that have not been widely approached or discussed in the literature, which has focused primarily on technical aspects of MPA network design e.g. ^20^. We acknowledge that the context for, and approaches to, conservation planning vary widely throughout the Coral Triangle. While some of the strategies we propose are being applied to some extent in parts of the region, and we highlight examples of these, without exception they are not being addressed effectively or extensively enough in the Coral Triangle, or indeed in many other regions. The overall goal of this paper is to contribute to the evolving agenda for marine conservation planning in the Coral Triangle by stimulating dialogue about these important and neglected topics.

**Table 1.** Summary of ten things to get right for marine conservation planning to effectively inform management actions in the Coral Triangle. Each of these topics is discussed further in the text.

Issue	Explanation	Key challenges	Suggested actions
1. Making conservation planning accessible	To be broadly applied, conservation planning needs to be accessible to a wider range of practitioners working in the region, including government agencies at levels from local to national	Increasing the exposure of those responsible for spatial planning and resource management to concepts and methods in conservation planning Dispelling misconceptions about conservation planning Demonstrating the benefits of planning, and costs of not planning	Develop locally-appropriate tools and approaches (i.e. that are not resource- intensive or software-dependent) Translate technical documents and case studies into local languages Document contextually-relevant case studies
2. Integrating conservation planning with other planning processes	Conservation plans must better integrate with the broader suite of marine spatial planning processes. This will avoid conservation being marginalised, conflicting unnecessarily with often more influential commercial decisions, and imposing avoidably on resource users with little financial or political power	Identifying how to interface with, and inject a conservation perspective into, other planning processes Explicitly identifying and reconciling trade-offs between objectives for conservation, commercial interests, and livelihoods	Improve integration within and between organisations responsible for aspects of marine spatial planning Reformat or refocus planning outputs to increase relevance to day-to-day decision making by diverse sectors Embed analysis of trade-offs within high- level decision making
3. Building local capacity for conservation planning	In-country capacity for conservation planning is essential for local ownership and long-term implementation of conservation plans	Fostering the broad skills-base required by conservation planners Broadening the base of in-country technical experts	Develop conservation planning short- courses and university curricula Develop qualifications and competency standards that recognise marine conservation planning as a profession
4. Institutionalising conservation planning within governments	Conservation planning must be established as a norm within government to avoid spatially restricted applications associated with project-based models, and to ensure that support for plans is sustained in the long-term	Diverse governance arrangements require context-specific approaches to institutionalisation Governmental reform typically requires long time-frames	Review the current legislative and institutional environment at different levels of government, to identify appropriate entry points
5. Integrating plans across governance levels	Conservation plans must be carefully integrated across spatial scales and levels of governance to avoid plans and policies at different levels that conflict, or are difficult to interpret or enforce	Overlapping legislation and unclear jurisdictions, often with multiple implementing government agencies and customary authorities at different levels Scale-dependence, whereby management initiatives depend on actions taken at higher or lower jurisdictional levels	Legal reform to ensure that plans consider existing laws and regulations at different scales Further develop the capacity of the Coral Triangle Atlas to track the contribution of local actions towards wider objectives
6. Planning across governance boundaries	Where management is decentralised, transboundary coordination will be necessary to avoid social-ecological scale mismatches, where the spatial extent of ecological processes exceeds that of management jurisdictions	Resolving inequitable distribution of conservation costs and benefits Aligning multiple, sometimes divergent, objectives within different governance units	Support efforts to develop local governance networks Explore innovative ways to overcome equity issues, e.g. payments for transboundary ecosystem services
7. Planning for multiple tools and objectives	Conservation planners have become proficient at designing networks of fully protected areas, but a wider range of locally relevant tools and approaches that can also achieve conservation goals should be considered	Cross-sectoral integration of goals related to biodiversity, fisheries and food security Better understanding the contribution of different management actions towards different objectives	Document case studies where conservation plans incorporate multiple zones or management tools Review the effectiveness of different management tools at ameliorating context-specific threats and achieving objectives
8. Understanding imitations of data	Whilst data limitations are unavoidable, conservation decisions can be made more effectively where the shortcomings of data can be understood or avoided	Non-nestedness of biodiversity priorities Discordance between the resolution at which conservation priorities are identified and at which they are useful to inform management	Modify collection of census data to include socio-economic metrics relevant to resource management Capitalise upon improved quality and availability of habitat data derived from remote-sensing Recognise that conservation plans will require updating as better data become available
9. Developing better measures of progress and effectiveness	Common measures of progress focus on outputs rather than outcomes, risking “residual” conservation actions that fail to achieve meaningful progress towards objectives	Changing the norm whereby extent of protected areas is equated, often mistakenly, with conservation progress	Conduct applied research to adapt and extend existing methods for evaluation of conservation impact to the Coral Triangle Ensure that established monitoring and evaluation programs produce data that can be used to assess impacts of conservation interventions
10. Making a long-term commitment	The long-term commitment required for effective conservation planning is under- appreciated: conservation planning must be conceived, and adequately funded, as a complete planning – implementation package	Overcoming mismatches between short-term funding and political cycles and long-term needs for planning and implementation	Shift from project-based conservation towards institutionalised processes and funding allocations for conservation planning Planning teams must learn to work more effectively within short-term funding cycles Donors must understand that conservation needs long-term funding, or more modest short-term objectives

## Things to get right

For each of our 10 topics (summarised in Table 1), we first outline a problem statement, and then suggest potential ways forward.

### Making conservation planning accessible

Historically, marine conservation planning in the Coral Triangle has typically been initiated and led by non-government organisations (NGOs) or academia, often through collaboration with local communities or governments ^18, 21– 23^. These planning initiatives, although valuable in demonstrating concepts, analyses and, at limited scales, applications to on-ground actions, have inevitably focused on specific areas within the Coral Triangle that constitute a very small proportion of the entire region. To achieve wider application of marine conservation planning, the established and emerging approaches and tools need to be made more accessible to a much wider range of practitioners, including those in government agencies responsible for spatial planning at levels from local to national.

Beyond a lack of local capacity to implement conservation planning methods (see “ Building local capacity for conservation planning”)., there exists a lack of awareness of what conservation planning is and why it is needed, among people responsible for managing coastal and marine resources. In the past, the dominance of developed countries in generating research on conservation planning biased approaches toward extensive prioritizations and, where these are actually applied, toward simple governance contexts ^7^. These biases led to earlier misconceptions amongst policy-makers and potential practitioners in developing countries that conservation planning is only relevant to top-down, centralised planning ^24^, that it is incapable of dealing with the social, economic, and cultural complexities of the Coral Triangle ^25^, and that planning processes depend upon the use of decision-support software and data of a quality and quantity that are generally unavailable in the region. The CTI-CFF has helped to reduce these misconceptions and recognise the importance of combining bottom-up and top-down engagement to achieve goals for conservation planning. For example, the Solomon Islands and Timor-Leste have embraced and allocated funding for community-based planning as the foundation of their national approaches (personal communication: R. Pinto, Conservation International Timor-Leste; A. Vavekaramui, Solomon Islands Ministry of Environment, Meteorology, Disaster Response and Climate Change). However, systematic conservation planning is still largely a peripheral and under-valued activity in the overall operations of government organisations.

Making conservation planning accessible requires increasing the exposure of those responsible for spatial planning and coastal resource management to conservation planning concepts and processes, and at the same time dispelling misconceptions that might have developed from limited information.

Much that is written about conservation planning appears in literature that is inaccessible to potential users in the Coral Triangle. A learning study conducted at the end of the five-year USAID Coral Triangle Support Partnership program ^26^ revealed that, among those surveyed, 54% never or rarely used the 265 separate knowledge products produced, and only 20% frequently or often used them. These products included position papers, books, training manuals, field guidance manuals and other materials produce by the USAID Coral Triangle Support Partnership, the US National Oceanic and Atmospheric Administration and the US CTI Support Program Integrator based in Bangkok. Poor uptake is likely due to lack of awareness of recently produced materials, and the need for more simplified materials for some applications. This clearly shows that coastal resource management practitioners in the Coral Triangle are in a nascent stage with respect to accessing literature and knowledge products supporting conservation planning.

A broader, more accurate awareness of conservation planning will be achieved by encouraging researchers to publish in open-access journals ^27^, translating technical documents and case studies into local languages, and distributing presentations and documents through peer-learning networks (e.g. the Philippine MPA Support Network). Knowledge products need to be more effectively distributed, including in local languages, and materials that are made available need to be in forms that match the needs and capacity of their target audience. Simplified and demystified information products are required to dispel misconceptions about conservation planning and highlight the balance between top-down and bottom-up planning.

Misconceptions that planning is necessarily a “top down” process can generate reluctance amongst stakeholders to engage. Case studies such as those presented in Game *et al.* ^18^ and Weeks and Jupiter ^28^ demonstrate that conservation planning tools and methods can be used as inputs for community-based decision making. Further examples that emphasise entire, participatory planning processes and place less emphasis on decision-support tools are required. NGO-led initiatives tend to be supported by expertise and funding rarely available to government agencies e.g. ^22^. Case studies that demonstrate how conservation planning can be undertaken within the financial, technical, and resource constraints typical of government agencies within the Coral Triangle are needed. These are now emerging; an example is the recently approved and budgeted community-based conservation program in Timor-Leste, piloted under the USAID CTSP program ^29^.

Finally, it will be necessary to demonstrate the benefits of planning, and costs of not planning, compared to counterfactual scenarios of unplanned expansion of MPAs or alternative management strategies ^19^. For example, it can be demonstrated that objectives for biodiversity conservation can be achieved at a lower cost to resource-users under planned than unplanned scenarios ^19^, and that opportunity costs to different users can be explicitly and transparently identified to minimise conflict ^30^.

### Integrating conservation planning with other planning processes

Coastal areas and inshore waters are subject to many potentially competing planning processes, such as those for maritime transport, environmental protection, energy, fisheries, and tourism. Frameworks for marine spatial planning and integrated coastal zone management have been proposed to integrate the spatial aspects of sectoral policies in these diverse areas ^31^. These frameworks aim to meet ecological, economic, and social objectives ^32^, facilitate explicit trade-offs between competing uses, improve transparency in decision-making, and help to avoid unnecessary conflicts ^33, 34^. Yet coastal resource management in the Coral Triangle remains highly sectoral, with overlapping and incompatible jurisdictions, and unclear, and sometimes conflicting, mandates for different government agencies ^35, 36^. Aside from avoiding areas obviously incompatible with conservation (e.g. ports, shipping lanes), there are few examples of fully integrated spatial plans.

Spatial and non-spatial planning strategies relating to production and development sectors are likely to be better funded, more widely understood, and more strongly institutionalised within government (see “ Institutionalising conservation planning within governments”) than conservation planning. Consequently, to have influence, conservation plans must interface with and inject a conservation perspective into these planning processes. This integration has been referred to as mainstreaming conservation plans ^37, 38^. Failure of conservation planners to engage with the larger enterprise of marine spatial planning involves several risks: marginalisation of conservation objectives; unnecessary conflict between conservation plans and more influential, development initiatives; and adverse, avoidable impacts on local communities reliant on marine resources for subsistence or small cash economies.

Several advances are needed to better integrate conservation planning with the diverse aspects of marine spatial planning. Influence of conservation thinking on development planning requires anticipating commencement or reviews of development plans and policies, sectoral integration between agencies responsible for conservation and development planning, strong liaison of conservation scientists and NGOs with agencies responsible for development planning, and appropriately formatted information to provide inputs to processes for planning development ^37^. Disparity between objectives can result in differences in structure and content between the outputs of conservation planning processes and those required for spatial planning more generally ^37^. Integration can be facilitated if decision-support software tools developed to address the problem of prioritising areas for biodiversity conservation (e.g. Marxan, Zonation, C-Plan) are refocused to guide strategic expansion of development or extractive activities to have minimal impact on high-value sites for biodiversity ^39– 42^. An example would be to delineate shipping channels to minimise impacts on marine megafauna.

Some progress toward sectoral integration is evident in the Coral Triangle. In Indonesia, prior to laws relating to spatial planning and management of coastal areas and small islands passed in 2007, coastal resources were governed by a vast array of statutes and laws with dozens of implementing agencies ^35^. Indonesia is now moving towards a more integrated approach ^43^. However, local government agencies previously mandated to zone terrestrial and urban areas lack capacity in marine conservation planning ^44^ (see “ Building local capacity for conservation planning”).

Ultimately, effective integration of conservation objectives and priorities into marine spatial planning requires explicit analysis of trade-offs: specifically, identifying the extent to which diverse objectives for conservation, development, and livelihoods are mutually exclusive, and providing a decision-making framework to resolve conflicts with a proper understanding of the implications of some objectives not being fully achieved ^45, 46^. The required methods are being devised, but have seldom been applied for real-world decisions, and remain inaccessible to emerging leaders in Coral Triangle countries.

### Building local capacity for conservation planning

Since the CTI-CFF was conceived, building capacity has been a priority for all six Coral Triangle countries, which are presently under-resourced to support the >1500 existing MPAs, let alone achieve ambitious goals of protecting 20% of marine and coastal habitats by 2020 ^12^. Even countries with relatively well-developed capacity for marine conservation planning - Malaysia, Philippines, and Indonesia ^47^ - have identified lack of in-country capacity as a key hurdle to achieving CTI-CFF goals ^36^.

Although training on MPA management is available (from NGOs, government, and universities), lack of communication and coordination between training providers has led to delivery of non-standard modules, duplication, and omission of key competencies ^36^. Furthermore, capacity building is often delivered as one-off training and, without follow-up assistance and mentoring, skills and knowledge acquired during training can be quickly lost. To undertake conservation planning, individuals, or at least planning teams, need to have a broad range of skills and knowledge that extend beyond those typically covered by existing training. Skills are needed in ecology, social science, the use of specialist software or GIS (geographic information systems), stakeholder engagement, communication, and negotiation, to name a few.

As a consequence of these limitations, managers of MPA networks depend heavily upon assisting organisations (e.g. NGOs, academe, development partners, or donors) for technical support with planning ^18, 48^. There are few organisations in the Coral Triangle with sufficient capacity to develop and implement effective conservation plans, and those that have the capacity are not sufficiently staffed or resourced to extend their support to all who request it. Broadening the base of technical experts will be crucial, especially in smaller countries such as the Solomon Islands, Timor-Leste, and Papua New Guinea, where existing experts are stretched to deliver support across the many problems requiring their attention ^49^.

A common approach to develop capacity in the region is through peer-learning networks, such as the Philippine MPA Support Network, Papua New Guinea Centre for Locally Managed Areas, the regionally focused Coral Triangle Center in Bali, Indonesia, and the Locally Managed Marine Areas network, active in Solomon Islands and Indonesia. These learning networks facilitate cross-site visits and similar events that allow members to share experiences and lessons learned, and provide access to training modules or events. Although training often focuses on specific aspects of MPA management, learning networks could provide a venue through which information on conservation planning might also be disseminated.

A crucial solution to develop capacity in the long-term will be to create a new cohort of conservation planners from the region, through development of specific courses, qualifications, and competency standards that recognise marine conservation planning as a profession ^50^. Targeting students in related disciplines with university short-courses and curricula ^51^ that focus specifically on marine conservation planning would be a short-term step in this direction. The University of the Philippines’ Marine Science Institute is currently developing a Masters program on Tropical Marine Ecosystem Management aimed at local government employees and MPA managers, which includes specialisations on MPAs and spatial planning. We envision that conservation planners will eventually be represented within the relevant national government agencies and local governments in the Coral Triangle region (see “ Institutionalising conservation planning within governments”).

Capacity might also be built through improved and sustained collaboration between scientists from developed nations and local research communities. For example, a Partnerships in International Research and Education (PIRE) project funded by the US National Science Foundation placed US graduate students and postdoctoral scholars in research institutions in Indonesia and Philippines for a year, providing improved laboratory infrastructure, research funding, and new educational opportunities for Filipino and Indonesian scientists and students ^27^. USAID has also funded partnerships between US and Indonesian universities, fostering strong connections between Indonesian scientists and international collaborators ^27^.

### Institutionalising conservation planning within governments

At present, conservation plans for regions within the Coral Triangle are frequently developed and implemented as projects led by NGOs or academic institutions with restricted time frames and limited budgets for engagement. Project-based conservation planning is undesirable for two reasons. First, supporting organisations have their own motivations for involvement in planning initiatives, which are manifest in the regions selected for planning effort: typically those with extraordinary biodiversity value or particular research interest ^12, 52^. Thus, under the project model, conservation planning is spatially biased and will be undertaken only in few parts of the region. Second, conservation plans quickly become out-dated as ecological and socioeconomic conditions change. In the common case of protracted implementation, continuity of resources and expertise is required over extended time periods ^7^. If plans are conceived and developed as finite projects, funds might not be secured for the ongoing implementation, adaptation, and revision required to keep them relevant, and personnel with capacity to interpret and update plans might be lost to lead organisations or redeployed to other roles. Institutionalising conservation planning within government will ensure that planning effort is invested much more widely, and is necessary if resources for protracted implementation and adaptation are to be maintained.

Institutionalizing conservation planning will present substantial challenges, but none appear to be intractable. Each of the six countries of the Coral Triangle has distinct governance arrangements with respect to spatial planning, biodiversity conservation, and management of coastal resources ^14^. Approaches to institutionalise conservation planning will therefore need to be sensitive to these differences. Within-country differences in approach will also be necessary ^47^. Governmental reform is seldom rapid, although the need to embed conservation planning in government at all levels is urgent. Still, the groundbreaking nature of the CTI-CFF itself, and the consequent progress toward multi-jurisdictional vertical (see “ Integrating plans across governance levels”) and horizontal (see “ Planning across governance boundaries”) cooperation, demonstrates that high-level reform for marine conservation is possible.

A practical first step towards institutionalizing conservation planning would be to review the current legislative and institutional environments, at different levels of government (including customary governance) in each country, to identify appropriate entry points at which authority, legitimacy, and willingness to undertake conservation planning overlap. For example, Indonesia has comprehensive legislation that requires district governments to prepare spatial plans. These same government units are responsible for implementing MPAs, and thus offer an entry point to integrate conservation planning perspectives (see “ Making conservation planning accessible”). In contrast, in Papua New Guinea, there is no formal legislation supporting declaration of MPAs or spatial planning. However, strong systems of traditional resource ownership and customary law ^53^ provide an alternative route by which conservation plans can be developed and implemented by communities with customary tenure ^18^, e.g. ^22^. Here, conservation planning might better be institutionalised within customary, rather than formal, governance structures.

### Integrating plans across governance levels

Levels of governance in the Coral Triangle range from international to national, sub-national (provinces, states), and local (e.g. municipalities, districts, communities). Decisions made at one level of governance influence the suite of actions available to, or mandated by, decision-makers at other levels ^54^. Thus, spatial plans must be carefully integrated across spatial scales and levels of organisation ^55, 56^ to avoid plans and policies that conflict, or are difficult to interpret or enforce.

Use of marine and coastal resources in the Coral Triangle is frequently subject to overlapping legislation and unclear jurisdictions, often with multiple implementing government agencies at different levels ^35, 57^. For example, in Indonesia, the enactment of a series of laws in 1999 shifted responsibility for spatial planning and coastal resource management from the national to the district level, leading to conflict with pre-existing laws and ambiguity regarding the roles and responsibilities of national, sub-national, and local government authorities ^35^. National, sub-national, and local governments’ roles typically address different public needs and consequently can have different, sometimes conflicting, perspectives ^58^. In many parts of the Coral Triangle, governance is further complicated by overlap of authority between formal and customary government systems: whilst customary tenure is recognised in national constitutions, traditional systems of natural resource management tend to be poorly integrated with national policies and legislation ^59^.

Integration of conservation plans across governance levels should operate in two directions. First, region-wide initiatives such as the CTI-CFF need to be supported by actions at national, sub-national, and local levels ^60^ by translating broad policy directives and planning principles into guidelines for identifying spatial priorities at progressively lower levels of governance. As seascape-scale planning initiatives become more common, there will be a need to ensure that these effectively inform local actions. This requires larger plans to be seen, not as static products, but as starting points for ongoing adaptation to changes in local circumstances, including unforeseen errors in seascape-scale data ^7^. Similarly, national policies must be reflected in local plans. For example, in Malaysia, national regulations spatially demarcate a “commercial fishing zone” beyond three nautical miles from the coastline and a “traditional fishing zone” within that limit; these regulations provided a foundation for the process of zoning the Tun Mustapha Park, which subdivided the “traditional fishing zone” ^12^.

Second, local marine management actions must be legally recognised and reinforced by higher levels of governance ^61^. This is necessary both for local-level legislation, and customary governance. Otherwise, rules conceived and implemented locally might not be enforceable to outsiders who do not respect local customs and are beyond the reach of community-imposed punitive actions ^62, 63^. A further challenge is to anticipate and keep track of local actions not planned for at higher governance levels and the contribution that these make towards wider objectives. The Coral Triangle Marine Protected Area System (CTMPAS) framework, supported by the Coral Triangle Atlas, will play an important role in facilitating this ^64^.

In some contexts, scale-bridging organisations and networks, such as the Solomon Islands’ Locally Managed Marine Areas Network and Philippines’ MPA Support Network, can play a critical role in facilitating interactions between levels of governance ^48, 65^. Other contexts might require legal reform, to ensure that plans consider existing laws and regulations at different scales ^66^. Later revisions of Indonesia’s decentralization framework, for example, sought to clarify jurisdictional roles by emphasizing relationships between national and district governments, rather than local autonomy ^35^.

Efforts to align policy across governance levels must be undertaken with care. Vertical interplay between management systems at different jurisdictional levels can be complicated by divergent priorities, dominance over of one level over another (e.g. through de jure vs. de facto authority, or control over allocation of financial resources), and preferred knowledge systems: local-level governance typically places high value on traditional knowledge and place-based insights, whereas regional governance institutions are more likely to draw upon western science ^67^. In Papua New Guinea, attempts to strengthen coordination between national and provincial fisheries authorities had the unintended consequence of weakening links with local governments, as provincial priorities became aligned with national interests (commercial fisheries) at the expense of local concerns ^58^.

### Planning across governance boundaries

The boundaries of natural resources rarely match those of the governance institutions responsible for managing them ^56, 68^. This is certainly true for marine resources in the Coral Triangle, where ecological connectivity processes can operate across spatial scales of tens to thousands of kilometres ^69, 70^, but where management is, for the most part, decentralised to local governments and communities ^71^. Where the scale of ecological processes exceeds that of management jurisdictions, transboundary coordination is essential to avoid management efforts being insufficient to adequately protect the features and processes concerned. Furthermore, some benefits from management, such as enhanced recruitment arising from protection of spawning aggregations, might be realised beyond the boundaries of managing jurisdictions, undermining support for management ^72^.

To achieve management outcomes across ecologically meaningful scales will require coordination of planning across governance boundaries ^68, 73^, as well as arrangements for equitable sharing of the costs and benefits of management ^74^. For example, if a fish spawning-aggregation site is protected in one jurisdiction, complementary seasonal restrictions on catch of that species in neighbouring jurisdictions can provide increased ecological and fisheries benefits in all jurisdictions ^75^. However, inequitable distribution of the costs and benefits of conservation among stakeholders or jurisdictions might result in social or political conflict, failure during implementation, or poor compliance with management regulations ^76, 77^. Plans that span multiple jurisdictions also need to incorporate multiple (sometimes divergent) objectives identified within different governance units ^78^.

Transboundary planning might be most easily approached at local scales. This has been achieved to some extent in the Philippines, through the formation of local government alliances for coastal resource management ^48^. Motivation for collaboration typically comes from recognition of a common resource base and shared threats, such as the intrusion of commercial fishing vessels into coastal waters ^79^. Where such a shared vision is absent, neutral assisting organisations can act as brokers, helping to overcome social or political obstacles to coordination ^80, 81^. Alternatively, more innovative approaches to transboundary coordination, such as payments for transboundary ecosystem services ^82^, might be required.

Planning across international boundaries is likely to present the greatest challenge. For example, achieving CTI-CFF goals on managing priority seascapes and ecosystem approaches to fisheries management will additionally require negotiating access to high-value shared stocks (e.g. tuna), issues of national sovereignty, and financing ^81^.

### Planning for multiple tools and objectives

Marine conservation planning has, to date, focused largely on the design and implementation of ‘no-take’ MPAs and MPA networks, although approaches that consider multiple actions are emerging in the literature e.g. ^83^. The establishment of a region-wide, comprehensive, ecologically representative, and well-managed CTMPAS is one of six strategic goals of the CTI-CFF ^4^, and guidelines for the size and location of no-take MPAs in the Coral Triangle have recently been developed ^20^. However, aside from the fact that few MPAs are presently well managed or adequately enforced ^14^, there are two important limitations of no-take MPAs as tools for biodiversity conservation in this region. First, where local dependence on resources is high, and spatial or occupational mobility is limited (as in much of the Coral Triangle), no-take zones are necessarily small. The median size of no-take areas in the Philippines, for example, is just 0.12 km ^2^ ^16^. Furthermore, in some areas of the Coral Triangle, tradition or preference for alternative management strategies means that permanent no-take areas are rarely supported by stakeholders ^72^. Second, whilst no-take MPAs have proven benefits for biodiversity, fisheries and food security, they cannot manage many threats to marine and coastal ecosystems, such as land-based sources of nutrients and sediment or coral bleaching events related to climate change, and they offer only limited protection for migratory and wide-ranging species but see ^23, 84^.

Furthermore, if conservation planning is to be relevant to the CTI-CFF, it must address not only MPAs but also cross-sectoral integration of goals related to biodiversity, fisheries, and food security, and help to resolve inevitable trade-offs between these e.g. ^2^. Part of this challenge is for conservation planning to move out of its comfort zone in designing networks of no-take MPAs to consider a wider range of coordinated management tools that can address all major threats at relevant scales ^85^. The need for conservation planning to address a broad suite of actions is underlined by some simple facts: 90% of coral reefs in the Coral Triangle are under threat ^3^, while >80% of the region’s coral reefs are likely to remain outside of the CTMPAS, and a large proportion of inshore reefs, whether inside or outside MPAs, are adversely affected by terrestrial runoff ^3^.

The Coral Triangle has a long history of employing traditional and customary management practices other than no-take MPAs. Examples are temporary or periodically harvested fisheries closures variously known as *sasi, tabu*, or *taboo*, ^63, 86^. Conservation plans that employ familiar strategies such as these will likely be better supported locally ^72^, and will fit within existing governance frameworks. Multiple-use zoning offers a more flexible approach to resource management that can help to resolve trade-offs between multiple objectives ^84^. For example, in Indonesia’s Nusa Penida MPA, multiple-use zoning was used to resolve conflict between marine tourism, seaweed farming, and fisheries activities, ensuring that the interests of all stakeholder groups were clearly represented in the plan ^12^.

Planning for multiple tools, zones, or objectives is more complex than designing no-take MPA networks for biodiversity conservation. It requires more parameters to be estimated (with inevitable errors), increasing the need for plans to be adjusted when errors become apparent during implementation ^7^, and requiring further iterations of planning and stakeholder consultations. For example, planning for multiple tools requires an understanding of the contribution of different management actions towards different objectives ^19^.

Ideally, conservation planning would extend from inland watersheds to offshore waters, with integrated management of coasts and near-shore marine ecosystems ^87^. Among the impediments to designing and implementing fully integrated land-sea planning is the need to work at multiple levels of governance (see “ Integrating plans across governance levels”) and across governance boundaries (see “ Planning across governance boundaries”). Although planning methods are extending into this complexity of geography and governance ^88^, practical applications of such integration in the Coral Triangle are rare.

### Understanding limitations of data

Limitations of data are unavoidable in conservation planning ^89^. These limitations apply not only to data on biodiversity, but also to data on costs, opportunities, threats, and other spatial variables that are increasingly being used to make spatial decisions ^7^. This is especially true in the Coral Triangle, where data are generally sparser than in some other regions ^71, 90, 91^. Whilst paucity of data should not necessarily be seen as an obstacle to initiating conservation planning processes, conservation decisions can be more effective in promoting the persistence of biodiversity and livelihoods if some important limitations of data are understood or avoided. We focus here on two aspects of mapped data: spatial resolution and surrogacy.

Spatial resolution refers to the size of the smallest homogeneous area that describes biodiversity, cost, opportunities, or threats. In general, the more extensive the coverage, the coarser is the spatial resolution of consistent data e.g. ^92^. This also means that fine-resolution data tend to be available only in small parts of many planning regions, if at all. One implication is that priorities based on coarse-resolution data can be poorly aligned to those based on fine-resolution data available over smaller extents ^93^. A related issue is that more extensive assessments tend to use larger planning units, sometimes even whole bioregions ^94^, thereby blurring spatial variation between management units (e.g. traditional fishing grounds), which are generally very small in the Coral Triangle ^71^, while also increasing estimates of overall conservation costs ^95, 96^. Discordance between the resolution at which priorities are identified and that required for decisions about on-ground management mean that extensive, coarse-resolution analyses have little to offer local managers ^54^. Importantly, there is no reason to assume that conservation priorities are spatially nested; very large planning units identified as priorities will not necessarily contain all the priority areas that would later be identified with smaller planning units ^7^.

Almost all data in conservation planning are surrogates, meaning that they approximate the variables of actual interest but for which spatial data are impossible to collect with available resources. Familiar examples are maps of ecosystems as surrogates for poorly mapped or still undescribed species ^8^. For threats, distance to population centres might be a surrogate for exposure of marine waters to destructive fishing practices, even though actual threats vary with types of fishing gear used, attitudes of local fishing communities, dependence on types of marine resources, and links to markets ^68, 97, 98^. With assessments that are more extensive and in regions with poorer data, conservation planning will rely on surrogates that are more remote from variables of primary importance, making priorities for conservation less reliable. In the Philippines, for example, coastal population density is strongly correlated with fishing pressure at the provincial scale but, at finer spatial resolutions, greater occupational diversity in more urbanised areas makes this a poor surrogate ^99^.

The most obvious solution to problems related to resolution and surrogacy of data is to collect more accurate information on variables of interest at the resolution of management units throughout the Coral Triangle. This is more easily said than done, of course, with about 800 coastal municipalities in the Philippines ^48^, and many thousands of management units across the Coral Triangle. Nonetheless, whilst recognising that investment in data might compromise investment in conservation actions, better data will eventually lead to better planning. Demonstrations of the prospects for improved data in the Coral Triangle include the increasing quality and availability of remote-sensing imagery on coral reefs e.g. ^100^, the potential to adjust collection of census information to improve socio-economic data for planning ^101^, and participatory mapping of resource use and features such as spawning aggregation sites, which has the added advantage of engaging local stakeholders in decisions about conservation.

In some cases, data and conservation assessments might simply have to be ignored because their use would be counterproductive. Data at very coarse resolution and based on unreliable surrogates will not only fail to resolve spatial variation relevant to applying actions, but can also pre-emptively divert attention from areas that would be identified as important, had better data been used. Similarly, very extensive conservation assessments that use large planning units can be counterproductive because two key (though generally implicit) assumptions are unreliable ^7^: uniformity (that priority is uniform within planning units); and nestedness (that high priorities at coarse resolution will contain all high-priority areas at fine resolution). These limitations mean that extensive prioritisations should be replaced with bottom-up assessments that build toward flexible regional designs.

### Developing better measures of progress and effectiveness

Conservation, whether for biodiversity or livelihoods, receives much attention globally through policy and legislation and large amounts of funding through diverse initiatives from governments, NGOs, and private donors. The objectives and performance of conservation initiatives, in the Coral Triangle and elsewhere, are measured mainly in terms of inputs (e.g. dollars invested), outputs (e.g. protected area extent), or, less commonly, outcomes (e.g. representation of marine ecosystems in protected areas). The widespread emphasis on outputs of marine conservation efforts is illustrated in the ongoing preoccupation with one of the internationally endorsed Aichi targets (Target 11, 10% of marine and coastal areas under protection). Similarly, the CTI-CFF *Regional Plan of Action* “ultimate target” is to include 20% of each major marine and coastal habitat type in strictly no-take replenishment zones ^4^.

The problem with these goals and measures is that outputs can be unrelated to progress for biodiversity conservation or livelihoods. For example, the extent of marine protected areas globally and in Australia reflects efforts made to establish them where they are most expedient politically and least required to protect biodiversity ^102^. In terms of livelihoods, there is little evidence that the extent of protected areas is related to benefits to people ^103^. Even outcomes can be poor measures of actual progress. For example, increases in representativeness, the number of ecosystems covered by protected areas, can mask simultaneous increases in the bias of protection away from those ecosystems most in need of protection ^104, 105^.

Measuring conservation progress in terms of inputs, outputs, and outcomes results in means (establishing protected areas) being confused with ends (making a positive difference for biodiversity or livelihoods). Fundamentally, marine protected areas and related management actions are intended to make a positive difference, yet this difference is almost never measured.

The emerging field of conservation impact evaluation ^106^ promises to enable funders and policy-makers to extend measures of progress and effectiveness to assess directly how much difference existing conservation actions make to biodiversity and livelihoods, or how much difference future actions could make. Impact evaluation measures the effects of an intervention by comparing what happened with the intervention compared with what would have happened without the intervention (i.e. the counterfactual; ^107^). It is important to note that impact evaluation of conservation initiatives is very distinct from environmental impact assessment of development projects.

Over and above measures of inputs, outputs, and outcomes, impact evaluation offers two critical improvements. The first is attribution – ensuring that the observed changes flow from the intervention being assessed, not from unrelated contextual changes ^108^. It is important, for example, to understand whether livelihoods improve in response to a conservation initiative, as distinct from increased living standards across a region related to, say, macroeconomic changes. The second improvement provided by impact over other measures is the distinction between means and ends ^6^. If the ultimate goal of a program is to reduce the loss of biodiversity, then impact is the amount of loss avoided, relative to the amount had the program had not been implemented. Approaches to measuring the impact of protected areas retrospectively, to provide lessons for the future, are now well developed ^109^. Approaches to predicting where future protected areas could have greatest positive impact are also available ^110^.

The existing work on impact evaluation of protected areas, although mostly focused on terrestrial ecosystems, can now be adapted and applied to marine conservation in the Coral Triangle. Following the lead of the health and energy sectors ^108^, impact evaluation can also be extended to diverse on-ground interventions, such as partial fisheries closures, and strategic interventions including legislation, policy, and education. For these changes to happen, one requirement is applied research to adapt and extend existing methods for impact evaluation to the Coral Triangle, accounting for available data, capacity and the diversity of social and governance contexts. The other need is for impact evaluation theory and methods to be made more accessible to policy makers and practitioners in the Coral Triangle (see “ Making conservation planning accessible”). A first step towards this is to ensure that established monitoring and evaluation programs produce data that can be used to assess impacts.

### Making a long-term commitment

The long-term commitment required for effective conservation planning is generally under-appreciated ^7, 111^. Temporal-scale mismatches arise where short funding or electoral cycles conflict with long-term planning needs ^56^, and there has been a tendency towards funding models that value short-term project outputs, such as the development of conservation prioritisations or plans on paper, over long-term, effectively implemented outcomes (see “ Developing better measures of progress and effectiveness”). This is likely driven in part by the ease of demonstrating fulfilment of project goals linked to outputs, as opposed to less tangible outcomes, such as increased capacity of communities to undertake adaptive management. Another crucial factor is the time taken for the conservation impact of investments to become manifest and the general lack of methods for measuring impact ^6^. Focusing on short-term outputs fails to recognise that spatial prioritisation is merely the first, and arguably the easiest, phase of conservation planning, and must be followed by protracted processes of application ^7^, monitoring, and ongoing adaptive management and planning. Failure to conceive, and adequately fund, conservation planning as a complete planning – implementation package is a major reason why plans have failed to find traction in many parts of the world ^111^. Approaches that acknowledge the need for application but allow insufficient time or funds might attempt to expedite implementation but, in doing so, risk losing the support of stakeholders, leading to poor compliance and failure.

Making a long-term commitment to conservation planning requires a single organisation with responsibility for steering planning outputs towards sustained outcomes. This will be realised through a shift from project-based conservation planning, towards planning processes institutionalised within government or NGOs (see “ Institutionalising conservation planning within governments”). This change in approach also requires a move away from project-oriented funding models by governments and donors towards institutionalised allocations for conservation planning that are increasingly embedded within government structures.

Whilst short-term political cycles are unlikely to change, opportunities might exist to safeguard conservation plans and actions against changes in political leadership or environmental orientation. At local governance levels, leadership and legislative processes tend to move more quickly than at higher levels, facilitating rapid implementation, but also allowing laws to be quickly revoked. One way to buffer against potential setbacks at the local level is to reinforce conservation plans through legislation at higher levels of government (see “ Integrating plans across governance levels”). This strategy was adopted for the Sumilon Marine Reserve in the Philippines after a newly-elected local mayor with links to commercial fishing operations actively sought to degazette the MPA ^112^. Another example is the new Solomon Islands National Protected Areas Act, which establishes a legal process for national recognition of sub-nationally established protected areas. There is a risk that formalising local conservation plans under national legislation can negate other benefits of localised governance, such as ownership and adaptive capacity e.g. ^113^, but this risk can be offset by transparency and participative processes.

Until long-term commitments to planning are accepted and adequately supported, planning teams dependent upon short-term funding cycles must learn to work more effectively within these constraints. For example, planning teams could communicate long-term objectives to donors and package constituent parts of the planning-implementation process as a sequence of stand-alone projects that appeal to donors, rather than focusing only on outputs or promising rapid progress to outcomes. Likewise, donors must understand that quick fixes and simplistic measures of success (see “ Making a long-term commitment”) can be counterproductive; conservation success needs long-term funding, or more modest short-term objectives as part of a longer sequence from plans to actions. Two critical needs are longer-term visions and realistic expectations of outcomes. These expectations might include capacity building, consolidating the effectiveness of existing conservation actions (not just establishing new ones), and other such activities that have less concrete or prestigious outputs, yet contribute towards meaningful outcomes.

Much was made of the huge scale of investment by international donors and NGOs at the inception of the CTI-CFF ( http://www.usaid.gov/global-waters/november-2010/coral-triangle). Yet achieving the Initiative's goals will take decades, and it is likely that the resources required to do this have been seriously underestimated. It was difficult for the architects of such an ambitious initiative to appreciate the full implications of its geographic and political scale, the complexity of resource-management challenges to be resolved, and the required building of capacity to ensure local ownership of plans and sustainability of management actions into the future. It was even more difficult for governments and private donors to commit funds for what was always to be a decades-long enterprise.

Only time will tell whether the CTI-CFF itself will secure the long-term commitment required at all scales and levels of governance to achieve lasting outcomes. There are the seeds of a single organisation to provide oversight and coordination in the CTI-CFF Regional Secretariat, currently hosted by Indonesia. Still in early stages, the Regional Secretariat has the potential to guide a shift from project-based conservation planning, towards planning processes institutionalised within the six CTI-CFF governments (see “ Institutionalising conservation planning within governments”). This will require leadership and organisations with conservation planning capacity at all scales and levels of governance.

## Conclusions

The challenges to successful implementation of conservation planning in the Coral Triangle are primarily related to issues of governance, capacity, knowledge flow, and communication. Although understanding of biodiversity patterns, processes, threats, and how to manage them continues to develop, current scientific knowledge is generally sufficient to develop effective conservation plans. Addressing the challenges discussed above will open the way for more sophisticated planning approaches, such as explicit incorporation of ecological connectivity.

Getting our ten things right for marine conservation planning will be difficult, and might seem overwhelming. But the first five years of the CTI-CFF have seen progress on multiple fronts that, for many observers, would have been unimaginable beforehand. In the right-hand column of Table 1, we highlight some immediate ways forward in resolving the challenges that we have reviewed. These ways forward require action, from researchers, governments, donors, and practitioners. Some suggested actions are challenges in themselves, and will require innovative and novel solutions.

Ideal conditions for governing environmental resources (and for conservation planning) are increasingly rare ^114^. Growing demand for natural resources and ecosystem services as a result of rapid human population growth ^3^, combined with widespread poverty ^59^ and corruption (within governments at all levels, and in other organisations) present significant obstacles to effective conservation of biodiversity in the Coral Triangle. Progress in resolving these wider issues will greatly improve the context within which conservation planning can be undertaken.

Other ways forward are more practicable. Present shortcomings in the application of marine conservation planning, such as the incompatible spatial scale of many conservation prioritisations, have contributed to misconceptions about the suitability of conservation planning generally and, specifically, its appropriateness in the Coral Triangle. Resolving these shortcomings conceptually, and demonstrably through contextually-relevant case studies, will help to overcome barriers to adoption of conservation planning approaches.

Nevertheless, whilst case-study prototypes and “best-practice” guidelines can be useful to encourage uptake of a new approach, planners working in the Coral Triangle must have the flexibility to develop strategies that are responsive to local needs and conditions ^60^, without needing to comply with standard approaches ^59, 115^. Governance, capacity, planning cultures, and traditions of management of natural resources vary widely within and among the Coral Triangle countries, so there will not be a “one size fits all” approach to conservation planning. Likewise, each of the challenges discussed above will play out differently, and assume different relative importance and urgency, in different geographies and contexts.

Perhaps the most important thing to get right, if conservation planning is to have real impact in the Coral Triangle, is a conceptual shift from conservation planning undertaken as a project, to planning undertaken as a process. Process-oriented planning commits agencies and stakeholders to long-term engagement, which is essential to transform conservation plans on paper into successful outcomes in the long-term. Increasingly, Coral Triangle governments are adopting leadership roles at different levels and scales, as reflected in increasing national and sub-national budget allocations for conservation planning. These leaders need direct support to ensure that emerging approaches and tools become institutionalised. Finally, conservation planning should not be considered as a new paradigm for the Coral Triangle, adding to the workload of conservation practitioners and government agencies charged with natural resource management. Instead, conservation planning can be correctly seen as a way of integrating the multiple goals of the CTI-CFF and diverse additional goals to which governments are already committed.

The challenges discussed here are likely to also be faced by other regional-scale initiatives to manage common environmental resources, such as the Western Indian Ocean Coastal Challenge ( http://www.wiocc.org) and the Micronesia Challenge ( http://www.micronesiachallenge.org). Our insights into both challenges and potential ways forward might also be usefully applied or adapted to these regions.
