# Supplementary material for: Ten things to get right for marine conservation planning in the Coral Triangle
Source: F1000Res. 2015 Dec 21;3:91. Originally published 2014 Apr 17. [Version 3] doi: 10.12688/f1000research.3886.3 (PMC4111118; doi:10.12688/f1000research.3886.3)
Supplement: Peer review history

# Peer review: Referee response for version 2

**DOI:** 10.5256/f1000research.4678.r5086

**Published:** 2014-07-18

**Authors:** Helen Yap

**Affiliations:** 1 The Marine Science Institute, University of the Philippines, Quezon City, Philippines

No further comment.

I have read this submission. I believe that I have an appropriate level of expertise to confirm that it is of an acceptable scientific standard.

# Peer review: Referee response for version 1

**DOI:** 10.5256/f1000research.4162.r4516

**Published:** 2014-05-15

**Authors:** Pedro Fidelman

**Affiliations:** 1 Sustainability Research Centre, University of the Sunshine Coast, Sunshine Coast, Qld, Australia

The paper, based mostly on expert opinion, identifies and discusses challenges to marine conservation planning in the Coral Triangle – a region where, despite pressing conservation issues, conservation planning has had relatively limited influence. The paper provides insights to inform management actions in the Coral Triangle that may also prove useful to other regions facing similar challenges.

Though the paper is of an acceptable standard, the authors may want to consider the following points:

**Introduction**

It would be beneficial to provide more details about what conservation planning is and why it is needed, and the complex setting (environmental, socio-economic and political) that characterise the Coral Triangle (see Fidelman *et al.*, 2012 ^1^). These would help contextualise the challenges identified in the following sections; many (if not most) of the challenges result, to some extent, from the complex nature of large-scale marine areas such as the Coral Triangle.

**Planning across governance boundaries**

This section addresses the problem of fit and scale; and would benefit from drawing on the corresponding scholarship (see e.g., Young, O.R., 2002 ^2^). The authors may want to consider replacing the heading “ *Planning across governance boundaries*” with “ *Addressing the problem of fit and scale*” or something along these lines.

**Conclusions**

It would beneficial to generalise the discussion beyond the Coral Triangle; that is, most of the challenges identified are also true to other regions, particularly large-scale marine regions. Insights from the paper would also be applicable to those regions.

**Other comments**

Some of the suggested actions to address the challenges identified are challenges themselves (e.g., legal reform, integration across governance levels, institutionalising conservation planning etc.). This is because we live in an increasingly complex world where (borrowing from Dietz *et al.*, 2003 ^3^) ideal conditions for governance (and for that matter conservation planning) are increasingly rare. That is, many of the challenges identified in the paper are facts of life; accordingly, the real challenge is to find innovative ways to navigate such challenges.   

Last, we address related challenges to the Coral Triangle in a recent article – Fidelman *et al.,* 2014 ^4^ – which the authors may find relevant to this paper.

I have read this submission. I believe that I have an appropriate level of expertise to confirm that it is of an acceptable scientific standard.

## References

[1] FidelmanPEvansLFabinyiMFoaleSCinnerJRosenF: Governing large-scale marine commons: Contextual challenges in the Coral Triangle. *Marine Policy*.2012;36(1) : 91 42-53 10.1016/j.marpol.2011.03.007

[2] YoungOR: The Institutional Dimensions of Environmental Change.2002; Reference source

[3] DietzTOstromESternPC: The Struggle to Govern the Commons. *Science*.2003;302(5652) : 91 1907-1912 10.1126/science.1091015 14671286

[4] FidelmanPEvansLSFoaleSWeibleCvon HelandFElginD: Coalition cohesion for regional marine governance: A stakeholder analysis of the Coral Triangle Initiative. *Ocean & Coastal Management*.2014;95: 91 117-128 10.1016/j.ocecoaman.2014.04.001

# Peer review: Referee response for version 1

**DOI:** 10.5256/f1000research.4162.r4520

**Published:** 2014-04-22

**Authors:** Helen Yap

**Affiliations:** 1 The Marine Science Institute, University of the Philippines, Quezon City, Philippines

This is a well-written paper. It summarizes valuable lessons learned from an extensive, not to mention expensive, conservation effort in a critical biodiversity-rich region of the world, the so-called "coral triangle".

My main comments addressed to the authors would pertain to the major obstacles that conservation initiatives have been confronted with over the decades, leading to very few success stories even to this day. I'm rather surprised they are not mentioned at all in the review.

The real issues, in my own experience, are poverty, human population growth, and corruption (both in the government level, but also in the NGO community).

Regarding the first issue, grinding poverty in the rural areas that abut coastal resources – not only in the countries of the coral triangle – appears to continue unabated, and is only exacerbated by dwindling natural resources, poor or negligent government, and the negative impacts of climate change (sea surface warming, drought, excessive precipitation, more violent storms). A vicious cycle ensues, as all informed individuals should know; namely, poverty begets more natural resource destruction, which begets more poverty… A desperate family (and there are millions of them) needing to put food on the table will catch the last fish, or fell the last tree, regardless of the best disseminated and best articulated conservation schemes at any level or spatial scale. If the status of biodiversity is to improve in the future, shouldn't this issue be addressed?

Which will probably take the authors to territory where they have little expertise, such as the local and global economic orders with their built-in inequities in terms of trade, fiscal policy, human migration, employment opportunities, etc. Though the term "macroeconomic" was mentioned at least once in the manuscript.

The second issue, that of human population growth, does not need much elaboration because it is a glaring one. However, it does have implications for the success of conservation campaigns, and at least deserves mention.

Finally, the issue of corruption. It’s a sensitive one, and I'm not sure the authors wish to tackle it head-on in case there are repercussions from national government or local officials they need to deal with in the course of their work. The matter of corruption within the NGO community I've heard about first-hand, such as the overcharging for boat use that is then passed on to the donors. Should such facts be taken into consideration when voicing concern about the success of conservation efforts in the long-term and, with them, the well-being of local human populations (e.g. "livelihoods") that they are supposed to help ensure?

As a minor comment, the paper might benefit from some graphs and other figures.

I have read this submission. I believe that I have an appropriate level of expertise to confirm that it is of an acceptable scientific standard, however I have significant reservations, as outlined above.

# Response

**Published:** 2014-05-13

**Authors:** Rebecca Weeks

**Affiliations:** Australian Research Council Centre of Excellence for Coral Reef Studies, Australia

Many thanks for your review Helen.

The issues that you raise - poverty, human population growth, and corruption – are certainly major obstacles to effective conservation of biodiversity, in the Coral Triangle and elsewhere.

Our aim with this manuscript was to highlight challenges specific to conservation planning, as opposed to those facing conservation initiatives more generally. This narrower focus led to our identification of ten challenges that are more directly related to the uptake and application of conservation planning and, we hope, more easily resolved through action by researchers and conservation practitioners, than the ones you mention.    

Of course, the context within which conservation planning is undertaken would be improved through efforts to resolve wider issues relating to poverty, population growth and corruption, and this warrants mention here.

In revision, we will both clarify the scope of the manuscript better, and include mention of the overarching challenges of population, poverty, and corruption. Please note though that we will await a second peer review report before revising our manuscript.
